# The interactive effects of oral health literacy and acculturation on dental care use among Hispanic adults

**DOI:** 10.1111/jphd.12529

**Published:** 2022-05-29

**Authors:** Daisy Patiño Nguyen, Michelle R. McQuistan, Fang Qian, Marcela Hernández, Mark D. Macek, Donald L. Chi

**Affiliations:** ^1^ Department of Oral Health Sciences University of Washington School of Dentistry Seattle Washington USA; ^2^ Department of Preventive and Community Dentistry The University of Iowa College of Dentistry Iowa City Iowa USA; ^3^ Iowa Oral Health Institute The University of Iowa College of Dentistry Iowa City Iowa USA; ^4^ Department of Family Dentistry The University of Iowa College of Dentistry Iowa City Iowa USA; ^5^ Department of Dental Public Health University of Maryland School of Dentistry Baltimore Maryland USA; ^6^ Department of Health Systems and Population Health University of Washington School of Public Health Seattle Washington USA

**Keywords:** acculturation, dental care, dental utilization, Hispanic, oral health literacy

## Abstract

**Objective:**

Assess whether there is an interactive effect between oral health literacy and acculturation on dental care use for Hispanic adults.

**Methods:**

Self‐identifying Hispanic adults participated in this observational study (*N* = 338). Oral health literacy (low vs. high) was measured using the Comprehensive Measure of Oral Health Knowledge (CMOHK). Acculturation (low vs. high) was measured using the Short Acculturation Scale for Hispanics (SASH) and operationalized a second way by the language in which the survey was completed (English or Spanish). The outcome was dental care use in the past year (yes/no). Confounder‐adjusted modified Poisson regression models were run to generate risk ratios (RR) and to test the hypothesis that participants with high oral health literacy and high acculturation would be more likely to have used dental care in the past year than participants with low oral health literacy and low acculturation.

**Results:**

About 65% of participants used dental care in the past year. The final models failed to show that participants with high oral health literacy and high acculturation were more likely to have used dental care than other participants. However, in the language proxy interaction model, participants with high oral health literacy and low acculturation were significantly more likely to have used dental care than participants with low oral health literacy and low acculturation.

**Conclusion:**

There may be an interaction between oral health literacy and acculturation when modeling dental care use for Hispanic adults that should be further explored.

## INTRODUCTION

Hispanics currently represent 18% of the U.S. population and comprise the largest minority group [1]. Hispanics have disproportionately higher rates of dental caries and periodontal diseases. National data from 2011 to 2012 indicate that the prevalence of untreated decay in Hispanic adults aged 20–64 was significantly higher (36%) than non‐Hispanic white adults (22%) [2]. This difference was also noted for adults aged 65 and older (27% for Hispanics vs. 16% for non‐Hispanic whites, respectively) [2]. Non‐Hispanic white adults aged 20–64 were also more likely to retain their teeth (51%) compared to Hispanic adults (45%) [2]. NHANES 2009–2014 data reveal that the prevalence of periodontitis among Mexican American adults and other Hispanic adults was significantly higher compared to non‐Hispanic white adults (60% and 49% vs. 37%, respectively) [3].

Dental care is an important contributor to oral health because it can aid in early diagnosis and treatment of oral diseases. Inequities in access to dental care may be one reason for persisting oral health disparities within the Hispanic adult population. Recent national data on yearly dental visits show that 58.5% of Hispanic adults aged 18–64 had a dental visit in the past year compared to 66.6% of white adults aged 18–64 [4].

There are multiple hypothesized facilitators of dental care use, including oral health literacy [5]. Oral health literacy is defined as one's ability “to obtain, process, and understand basic oral health information and services needed to make appropriate health decisions.” [6] Paasche‐Orlow and Wolf presented a model on hypothesized mechanisms that link health literacy and clinical outcomes: (1) access to care; (2) provider‐patient interactions; and (3) self‐care [7]. Furthermore, health literacy is influenced by factors like race and ethnicity, culture, and language—and previous work emphasizes the importance of understanding interactions among these variables [7]. Past work also indicates significant associations between health literacy and dental care use for adults, including those living in rural areas [8]. However, a systematic review found that among nine studies, the results were inconsistent regarding associations between oral health literacy and dental use [5].

Acculturation is another factor that may impact dental care use and is defined as the ability of an individual, usually from a racial or ethnic minority group, to adapt to the culture (e.g., beliefs, behaviors, and language) of the majority group [9]. While validated multi‐item acculturation scales exist, proxies such as language preference and length of time in the U.S., are more common because they are easier to administer [9]. Schumann et al. [10] conducted a systematic review and found that 57 different acculturation scales have been used in epidemiologic studies, with the validated Short Acculturation Scale for Hispanics (SASH) being one of the most common unidimensional scales. Language was used as a proxy for acculturation in 38% of the studies [10]. Because of heterogeneity within Hispanic populations, language preference alone may not be a sufficient indicator of acculturation [11].

Studies on acculturation and dental care use in Hispanics are mixed. A recent study reported a significant association between SASH scores and dental visits for Hispanic adults [12]. A review conducted by Tiwari and Albino reported that Latino adults who were more acculturated were more likely to obtain dental care for themselves and their children [13]. Stewart et al. [14] reported that Mexican‐American adults with high acculturation scores (≥5 on a scale developed by Cuellar et al. [15]) are more likely to use dental care than those with low acculturation scores (<5). However, studies by Jaramillo et al. [16] and Finlayson et al. [17] did not find that language preference or acculturation scores were associated with dental care use in the past 12 months among Hispanic adults.

Researchers have assessed oral health literacy and acculturation as independent determinants of dental care use, but have ignored potential interactions. As Carter‐Pokras and Behume [16] suggested, there is value in understanding interactions between acculturation and other variables. Determining whether an interaction exists between oral health literacy and acculturation is important for two reasons. This knowledge can lead to more accurate models of dental care use, and subsequently used to identify high‐risk subgroups that would benefit most from intervention efforts that are tailored to meet specific subgroup needs. The hypothesis tested in this study was that participants with high oral health literacy and high acculturation would be more likely to have used dental care than others, especially participants with low oral health literacy and low acculturation.

## METHODS

### Study design

This was a cross‐sectional observational study. Participants were recruited from January 2015 to March 2015 in eight central and eastern Iowa communities, representing metropolitan and non‐metropolitan areas with a large Hispanic population. Participant recruitment methods were via word of mouth, fliers, mass email communication, and presentations at churches and social service organizations that serve Hispanic populations. The following inclusion criteria were adopted: self‐identify as Hispanic, age 18 years or older, reside in Iowa at the time of the study, and able to provide informed consent. This study was approved by the University of Iowa Human Subject's Office (IRB#201409832).

### Survey development

A survey was developed for this study to assess oral health literacy, acculturation, and dental use (see Appendices S1 and S2). When available, existing survey instruments were used. Oral health literacy was measured using the Comprehensive Measure of Oral Health Knowledge (CMOHK), which is a validated oral health literacy questionnaire composed of 23 items that assesses knowledge on prevention and management of dental caries, periodontal disease, and oral cancer (Appendix S1) [18]. Acculturation was measured using the validated questionnaire Short Acculturation Scale for Hispanics (SASH), a 12‐item scale that measures several aspects of acculturation, including behavior, cultural values, and language [9]. Additionally, a proxy acculturation variable was based on the language in which the survey was completed (Spanish or English). Dental use was measured with an item from the National Health and Nutrition Examination Survey (NHANES): “How long ago was your last visit to a dentist, dental hygienist, or other dental care provider?” Questions on dental visit frequency, reason for dental visits, history of dental disease and current oral health status, and demographics (e.g., age, sex, dental insurance status, education, marital status, rurality) were also included.

The survey was available in English and Spanish. A native Spanish speaker on the study team translated the English survey into Spanish. The translated survey was then reviewed by five University of Iowa faculty members, who were also native Spanish speakers, for content validity and word choice. Lastly, the revised survey was pre‐tested with three Spanish‐speaking community members by focus group to obtain feedback on readability, flow, and word choice. These individuals varied based on their countries of origin, age, educational attainment, socio‐economic status, and gender. When there were disagreements, focus group participants and members of the research team discussed them until consensus was reached.

### Study procedures

The survey was administered in person. To address potential reading challenges, a member of the research team verbally administered the CMOHK in the participant's language of choice (Spanish or English). A two‐sided flip chart that faced both the interviewer and participant had one CMOHK question displayed at a time, which gave participants the opportunity to read the question while the interviewer asked it aloud. The interviewer recorded the participant's responses. Before completing the remaining survey items, participants were asked “How confident are you filling out medical forms by yourself?” [19] Participants who indicated “never,” “occasionally,” or “sometimes” were read the remainder of the survey while those who answered “often” or “always” could choose whether to complete the survey themselves or by having the survey read aloud. About 33.5% of participants had the remaining survey items verbally administered by a team member and 66.5% read and completed the survey on their own. Participants received a $15 gift card to Walmart or Target after completing the survey.

### Outcome variable

The outcome variable, dental use, was measured using the question “How long ago was your last visit to a dentist, dental hygienist, or other dental care provider?” Responses were dichotomized into dental use in the past year (yes/no).

### Independent variable

The independent variable was oral health literacy measured using the CMOHK [18]. Each question was assigned one point and was scored as correct or incorrect. The number of points were aggregated to assign a score of poor (0–11 points), fair (12–14 points), or good (15–23 points) oral health literacy. Consistent with previous studies [20], the variable was dichotomized at the median into low (0–14 points) versus high (15–23 points) literacy.

### Effect modifier

Two separate measures of acculturation were adopted. The first acculturation measure was the SASH [9]. Answers to questions were scored on a five‐point scale, summed, and divided by 12 to obtain a score ranging from 1 to 5. Original scoring methods were used to determine acculturation levels. A score of 1–2.99 reflects lower acculturation while a score of 3–5 reflects higher acculturation. The second acculturation measure was the language in which the questionnaire was completed (Spanish vs. English). With this proxy measure, low acculturation was considered if the questionnaire was completed in Spanish while those who completed it in English were considered to have high acculturation.

### Model covariates

Based on the literature, six variables were hypothesized as potential confounders: age [17] (continuous), sex [21] (male vs. female), current dental insurance status [22] (none vs. other vs. private), education [23] (<12th grade vs. high school degree vs. some college or college degree), current marital status [24] (single vs. married/partnered vs. other), and rurality [25] (lives in a non‐metropolitan vs. metropolitan community).

### Modeling approach

A two‐step modeling approach was used. First, all potential confounders were identified (described above) to ensure completeness of the initial conceptual model. Second, for the analytics, only confounders that were statistically significant (*α* = 0.05) in the bivariate analyses were included in the models to ensure model parsimony (see below).

### Data analyses

Descriptive statistics were generated for the study population. Concordance was assessed between the two acculturation measures (SASH and language) using the Chi‐square test. The Chi‐square test and Wilcoxon rank‐sum test were used to examine bivariate associations between model covariates and dental use. Covariates that were significantly associated with dental use, oral health literacy, and acculturation were included in the final regression models as confounders (*α* = 0.05). Modified Poisson regression models were used to generate risk ratios and corresponding 95% confidence intervals (CI) to test the hypothesis that there would be an interaction between oral health literacy and acculturation on dental utilization. Poisson regression models provide unbiased estimates of risk ratios [26]. SAS for Windows (v9.4, SAS Institute Inc., Cary, NC, USA) was used for the data analyses.

## RESULTS

A total of 338 participants participated in the study (Table 1). The mean age of participants was 36.5 years (SD = 12.5; range: 18–71 years). Most participants were female (67.3%). About 39.9% of participants reported having private dental insurance, 37.3% reported no dental insurance, and 13% reported having Medicaid.

**TABLE 1:** Descriptive characteristics of Hispanic adults in Iowa and bivariate associations with dental use in the past year

Characteristics		Outcome: Used dental care in the past year		
	All participants *N* = 338	Yes *n* = 217N (%)	No *n* = 118N (%)	*p*‐Value
Age (years)				0.08
Mean ± SD	36.5 ± 12.5	37.2 ± 12.9	34.9 ± 11.9	
Sex (%)				0.03*
Male	110 (32.5)	61 (57.0)	46 (43.0)	
Female	224 (66.3)	154 (68.7)	70 (31.3)	
Missing	4 (1.2)	‐	‐	
Dental insurance status (%)				<0.01*
None	126 (37.3)	51 (40.4)	75 (59.5)	
Other (e.g., Medicaid)	44 (13.0)	35 (79.5)	9 (20.4)	
Private	135 (39.9)	116 (87.8)	16 (12.1)	
Missing	33 (9.8)	‐	‐	
Highest level of education (%)				0.06
<12th grade	103 (30.5)	62 (60.1)	41 (39.8)	
High school degree/GED	118 (34.9)	72 (61.0)	46 (38.9)	
Some college/College degree	110 (32.5)	81 (73.6)	29 (26.3)	
Missing	7 (2.1)	‐	‐	
Marital status (%)				0.51
Single	111 (32.8)	70 (63.0)	41 (36.9)	
Married/Partnered	194 (57.4)	131 (67.5)	63 (32.4)	
Other (Separated, Divorced, Widowed)	26 (7.7)	15 (57.6)	11 (42.3)	
Missing	7 (2.1)	‐	‐	
Rurality (%)				0.35
Non‐metropolitan	107 (31.7)	72 (67.3)	35 (32.7)	
Metropolitan	186 (55.0)	115 (61.8)	71 (38.2)	
Missing	42 (13.3)	‐	‐	
Oral Health Literacy (CMOHK) (%)				<0.01*
Low (0–14)	166 (49.1)	93 (56.0)	73 (43.9)	
High (15–23)	161 (47.6)	120 (74.5)	41 (25.4)	
Missing	11 (3.3)	‐	‐	
Short Acculturation Scale for Hispanics (SASH) (%)				0.05
Low (1–2.99)	240 (71.0)	147 (62.0)	90 (37.9)	
High (3–5)	93 (27.5)	68 (73.1)	25 (26.8)	
Missing	5 (1.5)	‐	‐	
Language in which survey was completed (%)				0.01*
Spanish	228 (67.5)	136 (60.1)	90 (39.8)	
English	109 (32.2)	81 (74.3)	28 (25.6)	
Missing	1 (0.3)	‐	‐	

^a^ Statistically significant using chi‐square test (*α* = 0.05).

Approximately 3.6% of participants reported never using dental care in the past year. Most participants (64.8%) reported dental use in the past year while 31.6% of participants reported using dental care more than 1 year ago.

The mean oral health literacy score was 14 (SD = 4.17; range: 0–22). About 49.1% of participants scored low on the CMOHK (<15 points) and 47.6% scored high (≥15 points).

Based on the SASH, 71% of participants had low acculturation. Similarly, the majority (67.5%) of participants completed the survey in Spanish compared to 32.2% who completed it in English. There was a significant association between SASH and language (*p* < 0.01; Table 2), with discordance observed for 12.7% of participants. Oral health literacy was significantly associated with both measures of acculturation (Table 3). Larger proportions of individuals with high oral health literacy (CMOHK) had high acculturation scores (SASH) than those with low oral health literacy (38.1% vs. 17.6%; *p* < 0.01). Similarly, larger proportions of individuals with high oral health literacy completed the survey in English than those with low oral health literacy (46.0% vs. 20.2%; *p* < 0.01).

**TABLE 2:** Agreement between two ways of measuring acculturation (SASH and language)

	Short acculturation scale for Hispanics (SASH) level		
	Low *n* = 239 *n* (%)	High *n* = 93 *n* (%)	*p*‐Value
Language in which survey was completed			<0.01*
Spanish	211 (93.78)	14 (6.22)	
English	28 (26.17)	79 (73.83)	

^a^ Statistically significant using chi‐square test (*α* = 0.05).

**TABLE 3:** Association between oral health literacy, as measured by the CMOHK, and two measures of acculturation (SASH and language)

	Comprehensive measure of oral health knowledge (CMOHK)		
	Low *n* (%)	High *n* (%)	*p*‐Value
Short Acculturation Scale for Hispanics (SASH)			<0.01*
Low	136 (82.4)	99 (61.9)	
High	29 (17.6)	61 (38.1)	
Language in which survey was completed			<0.01*
Spanish	134 (79.8)	87 (54.0)	
English	34 (20.2)	74 (46.0)	

Abbreviations: CMOHK, comprehensive measure of oral health knowledge; SASH, short acculturation scale for Hispanics.

^a^ Statistically significant using chi‐square test (*α* = 0.05).

In the bivariate analyses to identify confounders, sex, dental insurance status, oral health literacy, and language preference were significantly associated with dental care use in the past year (Table 1). For example, 68.7% of female participants reported using dental care in the past year compared to 57.0% of male participants (*p* = 0.03). A significantly larger proportion of participants with private dental insurance used dental care in the past year than the proportions of participants with Medicaid or other dental insurance and individuals without dental insurance (87.8% vs. 79.5% vs. 40.4%, respectively; *p* < 0.01). Furthermore, a significantly larger proportion of participants with high oral health literacy used dental care in the past year than participants with low oral health literacy (74.5% and 56.0%, respectively; *p* < 0.01). Larger proportions of participants who completed the survey in English used dental care in the past year than the proportion of participants who completed the survey in Spanish (74.3% vs. 60.1%; *p* = 0.01).

Additional bivariate analyses indicated that sex and dental insurance status were significantly associated with oral health literacy and both measures of acculturation. Specifically, while larger proportions of female participants had high oral health literacy than male participants (53.0% vs. 40.2%; *p* = 0.03), significantly smaller proportions of females scored high on the SASH (24.4% vs. 35.2%; *p* = 0.04) or completed the survey in English (28.6% vs. 39.5%; *p* = 0.04) compared to males. Significantly larger proportions of participants with private insurance had high oral health literacy (63.9%) compared to individuals with Medicaid or other dental insurance (54.6%) and individuals without insurance (36.4%; *p* < 0.01). Furthermore, larger proportions of participants with private dental insurance had high acculturation as measured by the SASH (45.1%) compared to participants with Medicaid or other insurance (29.6%) and without dental insurance (8.0%) (*p* < 0.01). Similar findings were observed by the language in which the survey was completed.

Based on the bivariate analyses, sex and dental insurance status were included in the models as confounders.

When assessed as main effects, there was no significant association between oral health literacy and dental use (Table 4). Similarly, both measures of acculturation were not associated with dental use.

**TABLE 4:** Interaction terms in modified Poisson regression models^a^ of oral health literacy (CMOHK) and acculturation on having used dental care in the past year

	Interaction model 1 CMOHK x acculturation (SASH) *N* = 289		Interaction model 2 CMOHK x acculturation (language) *N* = 292	
Variables	Risk ratio (95% CI)	*p*‐Value	Risk ratio (95% CI)	*p*‐Value
Oral Health Literacy (CMOHK)				
Low	Reference		Reference	
High	1.08 (0.94–1.26)	0.25	1.04 (0.91–1.20)	0.50
Short Acculturation Scale for Hispanics (SASH)				‐
Low	Reference		‐	
High	0.88 (0.76–1.01)	0.09	‐	
Language in which survey was completed		‐		
Spanish	‐		Reference	
English	‐		0.95 (0.83–1.09)	0.52
CMOHK x Acculturation (SASH)		0.53		‐
Low x Low	Reference		‐	‐
Low x High	0.92 (0.73–1.16)	0.52	‐	‐
High x Low	1.14 (0.95–1.36)	0.14	‐	‐
High x High	0.96 (0.79–1.17)	0.72	‐	‐
CMOHK x Acculturation(Language)		‐		0.03^b^
Low x Spanish	‐	‐	Reference	
Low x English	‐	‐	1.11 (0.89–1.38)	0.32
High x Spanish	‐	‐	1.22 (1.01–1.46)	0.03
High x English	‐	‐	1.00 (0.81–1.22)	0.98

^a^ Regression models adjusted for confounders (sex and dental insurance status).

^b^ Statistically significant (*α* = 0.05).

In terms of interactions between oral health literacy and acculturation as measured by the SASH, there was no significant difference in dental use for participants with high oral health literacy and high acculturation compared to participants with low oral health literacy and low acculturation (Table 4). However, in the language proxy interaction model, participants with high oral health literacy and low acculturation (i.e., Spanish) were significantly more likely to have used dental care in the past year than participants with low oral health literacy and low acculturation.

## DISCUSSION

To our knowledge, this study is the first to examine the interactive effects of oral health literacy and acculturation on dental care use for Hispanic adults. Contrary to our hypothesis, Hispanic adults with high oral health literacy and high acculturation were not more likely to have used dental care in the past year than those with low oral health literacy and low acculturation. In the language proxy model only, participants with high oral health literacy and low acculturation were significantly more likely to have visited a dentist in the past year than participants with low oral health literacy and low acculturation. These findings suggest that there are potentially important interactive effects between oral health literacy and acculturation in modeling dental care use for Hispanic adults.

Although no other studies have examined the interaction we tested, our study was guided by existing evidence suggesting that oral health literacy and acculturation are each independent determinants of dental care use [14, 22]. Our null findings may be related to study methodology, specifically in terms of the literacy and acculturation measures that were adopted. For example, our literacy measure (CMOHK) assessed one aspect of literacy (knowledge) and did not capture other elements of literacy like reading comprehension and communication skills [6] that may be related to dental care use. Future research should develop comprehensive measures of oral health literacy, which currently do not exist in dentistry, and validate them in Hispanic populations.

In addition, both acculturation measures had limitations. Language is a proxy for acculturation and SASH is a unidimensional scale [9]. Past work indicates that proxy measures are insufficient in addressing heterogeneity within the Hispanic population [16] and that multidimensional scales are more appropriate in modeling acculturation [9]. Furthermore, the null main effects findings introduce the possibility that other factors related to acculturation, including discrimination, structural racism, and access to resources, may be more important determinants of dental care use for Hispanic adults [27, 28]. Future research should continue to study these factors with an emphasis on refining conceptual models that include appropriate measures of acculturation, if relevant, to inform clinical intervention development.

In the language proxy regression model, participants with high oral health literacy and low acculturation were significantly more likely to have used dental care in the past year than participants with low oral health literacy and low acculturation. These findings suggest there is an interaction between oral health literacy and acculturation in modeling dental care use for Hispanic adults, but not in the originally hypothesized direction. There was no indication of any significant interactions in the SASH regression model. Definitive explanations are beyond the scope of the current study, but our findings provide a foundation for additional research aimed at further understanding these relationships.

In terms of intervention relevance, future efforts may need to account for both oral health literacy and acculturation to address disparities in dental case use that persist among Hispanic adults. A potential starting point is to focus efforts to improve dental use on individuals with low oral health literacy, regardless of degree acculturation, as well as Hispanics with high literacy and a preference for English, which accounts for findings from our language proxy interaction model. Community‐based participatory research methods could be used to identify the specific barriers to dental care that affect these individuals and to subsequently develop pilot interventions aimed at improving dental care use [13]. Provider‐level barriers to dental care use should also be identified to ensure sustained dental care use. Potential barriers include insufficient capabilities of the dentist and office staff to communicate with patients with low literacy, discriminatory office practices, and mistrust [29, 30].

There are at least four study limitations. First, this was a cross‐sectional study. Because acculturation is dynamic, longitudinal assessment of acculturation as a determinant of dental care use for Hispanic adults is needed in future studies. Second, the study was based on a convenience sample with a relatively small N, and thus the analyses are likely underpowered, and most participants were recruited from churches and social service organizations that serve Spanish speaking populations, thereby limiting generalizability. Third, the CMOHK tool by Macek et al. [18] required translation for use with our Spanish speaking population. The CMOHK may require more extensive psychometric testing and refinement. Fourth, the outcome did not specify the reason for dental use (e.g., preventive vs. other type of visit). About 60% of participants stated that they use dental care regularly, 30% use dental care only when they are experiencing a problem, and 10% for emergency care. Future work should specify the type of dental use to help guide appropriate intervention development.

## CONCLUSION

Our findings suggest that there is an interaction between oral heath literacy and acculturation in modeling dental care use for Hispanic adults. Understanding such interactions are critical in helping researchers identify the highest need subgroups among vulnerable populations and develop appropriate interventions aimed at meeting the needs of such subgroups.

## Supporting information


**Appendix S1** Supporting Information

**Appendix S2** Supporting Information
