# Supplementary material for: The interactive effects of oral health literacy and acculturation on dental care use among Hispanic adults
Source: J Public Health Dent. 2022 May 29;82(3):295–302. doi: 10.1111/jphd.12529 (PMC9546387; doi:10.1111/jphd.12529)
Supplement: Supplementary file 1 — Appendix S1 Supporting Information [file JPHD-82-295-s001.docx]

Has a doctor or dentist ever told you that you had tooth decay?

1. Yes
2. No
3. I don’t know

Has a doctor or dentist ever told you that you had periodontal disease?

1. Yes
2. No
3. I don’t know

Has a doctor or dentist ever told you that you had mouth cancer?

1. Yes
2. No
3. I don’t know

Have you lost all of your upper and lower natural permanent teeth?

1. Yes
2. No
3. I don’t know

What is another name for the roof of your mouth?

1. Gingiva
2. Canine
3. Palate
4. Gland
5. I don’t know

This picture shows the inside of a person’s mouth. The arrow points to something hanging from the back of the throat. What is this structure called?


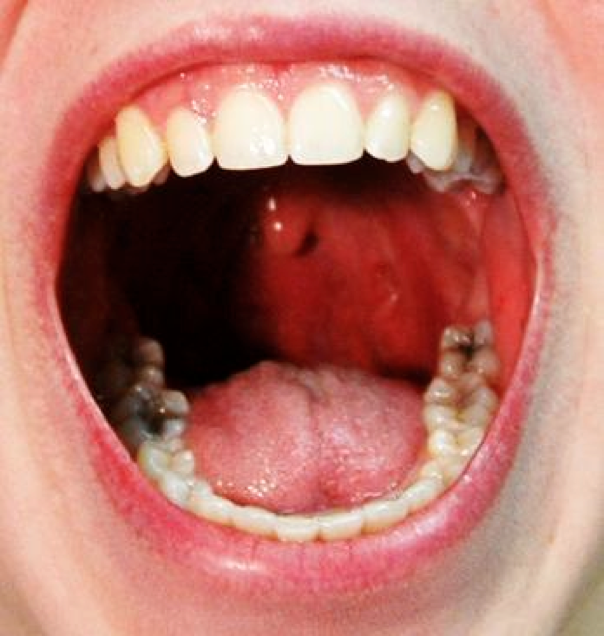


1. Incisor
2. Tonsil
3. Sinus
4. Uvula (YEWV-you-la)
5. I don’t know

How many **baby teeth** does a child **usually** get?

1. 10
2. 20
3. 32
4. 45
5. I don’t know

How many **permanent teeth** does an **adult usually** get?

1. 10
2. 20
3. 32
4. 45
5. I don’t know

How old are children when they get their **first adult** tooth?

1. About 1 year old
2. About 3 years old
3. About 6 years old
4. About 13 years old
5. I don’t know

As you understand it, what is the **main** purpose of braces?

1. Replacing missing teeth
2. Preventing tooth decay
3. Making teeth whiter
4. Straightening crooked teeth
5. I don’t know

As you understand it, what is the **main** purpose of adding fluoride to the public drinking water?

1. It kills germs in the water
2. It makes the water taste better
3. It protects teeth from tooth decay
4. It protects teeth from gum disease
5. I don’t know

As you understand it, what is the **main** purpose of dental implants?

1. Replacing missing teeth
2. Preventing tooth decay
3. Making teeth whiter
4. Straightening crooked teeth
5. I don’t know

This picture shows different parts of a tooth. To what part of the tooth is the arrow pointing?


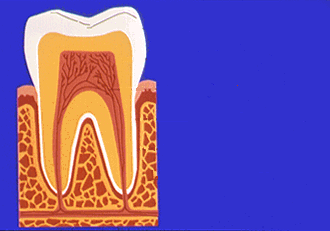


1. Incisor
2. Dentin
3. Premolar
4. Enamel
5. I don’t know

According to the American Dental Association, how often should adults **who have their own teeth** visit the dentist?

1. Every month
2. Two times per year
3. One time per year
4. When they have a toothache
5. I don’t know

In order to prevent tooth decay, people should avoid food with a lot of which of the following?

1. Salt
2. Spices
3. Fat
4. Sugar
5. I don’t know

What is the **main** reason infants should not be put to bed with a bottle that contains fruit juice?

1. The child’s teeth might not come in at the

right time

1. The child might get gum disease
2. The child might get tooth decay
3. The child might get crooked teeth
4. I don’t know

What is the **best** way a person can prevent tooth decay **at home**?

1. Using a toothpick after every meal
2. Drinking sugar-free soda
3. Rinsing with a mouthwash like

Listerine

1. Brushing and flossing every day
2. I don’t know

When a person has a **small** cavity, how does the dentist usually treat it?

1. Prescribing antibiotics
2. Placing a filling in the tooth
3. Pulling the tooth
4. Adding a dental implant
5. I don’t know

When a person has a **large** cavity, sometimes he or she needs a root canal. Which of the following describes what a root canal is?

1. Removing the tooth enamel
2. Removing the tooth dentin
3. Removing the tooth nerve
4. Removing the tooth cusp
5. I don’t know

This picture shows the inside of a child’s mouth. What do you think is wrong?

1. Gum disease

1. Tooth decay
2. Cold sores
3. Mouth cancer
4. I don’t know

This picture shows some gums that are puffy and red. What do you think this condition is called?


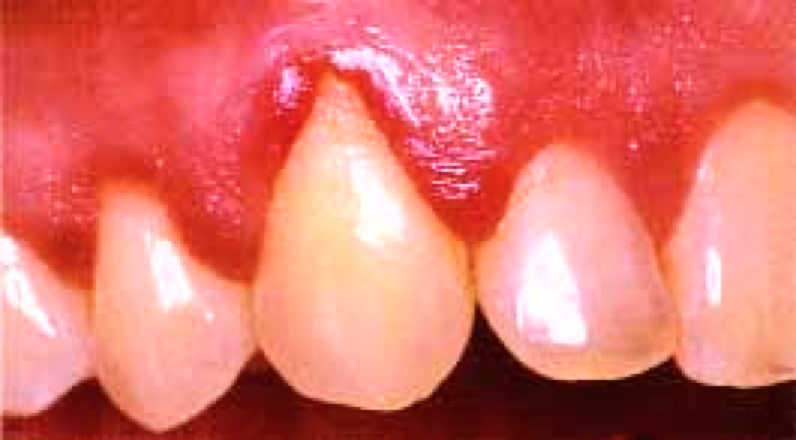


1. Gingivitis
2. Periodontitis
3. Canker (KAYN-ker) sores
4. Leukoplakia (Lou-ko-PLAY-kia)
5. I don’t know

Which of the following behaviors may cause periodontal disease?

1. Biting your fingernails
2. Eating spicy foods
3. Drinking too much coffee
4. Smoking cigarettes
5. I don’t know

Which of the following is the **best** way to remove tartar from a person’s teeth?

1. Eating hard foods like apples
2. Rinsing with a mouthwash like Listerine
3. Brushing and flossing
4. Getting a dental cleaning
5. I don’t know

This picture shows some teeth with receding gums. What do you think this condition is called?


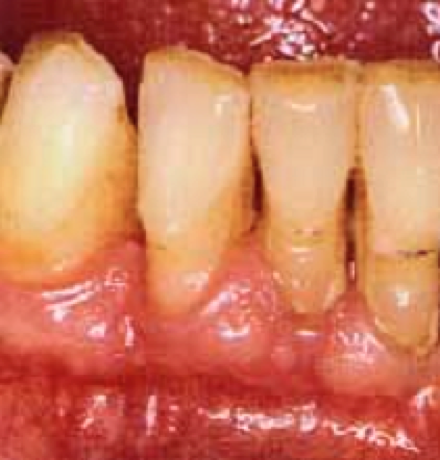


1. Fluorosis
2. Periodontal disease
3. Halitosis (hal-i-TOE-sis)
4. I don’t know

Periodontal disease is **more** likely to occur in people with which of the following conditions?

1. High cholesterol
2. Hepatitis
3. High blood pressure
4. Diabetes
5. I don’t know

What is the **most** common sign of cancer inside the mouth?

1. A sore that last more than two weeks
2. Pain when you open your mouth
3. Gums that bleed when you brush
4. Teeth that have black spots on them
5. I don’t know

Which of the following groups is **most** likely to get cancer inside their mouth?

1. Men younger than 40 years of age
2. Women younger than 40 years of age
3. Men older than 40 years of age
4. Women older than 40 years of age
5. I don’t know
