# Supplementary material for: The interactive effects of oral health literacy and acculturation on dental care use among Hispanic adults
Source: J Public Health Dent. 2022 May 29;82(3):295–302. doi: 10.1111/jphd.12529 (PMC9546387; doi:10.1111/jphd.12529)
Supplement: Supplementary file 2 — Appendix S2 Supporting Information [file JPHD-82-295-s002.docx]

ID#:___________________

Date: __ __ - __ __ - __ __

m m d d y y

Location:____________________

Participant Age: ___ ___ years

Participant Gender:

1. Female
2. Male

Survey completed by:

Survey read to participant in:

1. Spanish
2. English
3. Spanish and English

**Comprehensive Measure of Oral Health Knowledge will be administered first**

Answers:

| c1. | c5. | c9. | c13. | c17. | c21. | c25. |
| --- | --- | --- | --- | --- | --- | --- |
| c2. | c6. | c10. | c14. | c18. | c22. | c26. |
| c3. | c7. | c11. | c15. | c19. | c23. | c27. |
| c4. | c8. | c12. | c16. | c20. | c24. |  |

Oral Health Knowledge Score: ______________

Knowledge start time: ____________________

Knowledge finish time: ____________________

1. How confident are you filling out medical

forms by yourself?

1. Never (0%)
2. Occasionally (25%)
3. Sometimes (50%)
4. Often (75%)
5. Always (100%)
6. In the following situations, which language(s) do you prefer to use: **only Spanish (1), Spanish better than English (2), both equally (3), English better than Spanish (4), or only English (5)?**

|  | **Only Spanish** | **Spanish better than English** | **Both equally** | **English better than Spanish** | **Only English** |
| --- | --- | --- | --- | --- | --- |
| 1. In general, what language(s) do you read and speak? | 1 | 2 | 3 | 4 | 5 |
| 1. What was the language(s) you used as a child? | 1 | 2 | 3 | 4 | 5 |
| 1. What language(s) do you usually speak at home? | 1 | 2 | 3 | 4 | 5 |
| 1. In which language(s) do you usually think? | 1 | 2 | 3 | 4 | 5 |
| 1. What language(s) do you usually speak with your friends? | 1 | 2 | 3 | 4 | 5 |
| 1. In what language(s) are the TV programs you usually watch? | 1 | 2 | 3 | 4 | 5 |
| 1. In what language(s) are the radio programs you usually listen to? | 1 | 2 | 3 | 4 | 5 |
| 1. In general, in what language(s) are the movies, TV, and radio programs you prefer to watch and listen to? | 1 | 2 | 3 | 4 | 5 |

For the following questions, please circle the category that best describes your social preference: **All Latinos/Hispanics (1), More Latinos than Americans (2), About half and half (3), More Americans than Latinos (4), or All Americans (5).**

|  | **All Latinos/Hispanics** | **More Latinos than Americans** | **About half and half** | **More Americans than Latinos** | **All Americans** |
| --- | --- | --- | --- | --- | --- |
| 1. Your close friends are: | 1 | 2 | 3 | 4 | 5 |
| 1. You prefer going to social gatherings/parties at which the people are: | 1 | 2 | 3 | 4 | 5 |
| 1. The persons you visit or who visit you are: | 1 | 2 | 3 | 4 | 5 |
| 1. If you could choose your children’s friends, you would want them to be: | 1 | 2 | 3 | 4 | 5 |

**Behavioral and Cultural Total**: _­___/­12=_­___

**Language Proficiency (a,c,d,e) Total:**_­___/4=__­___

1. In which town or city do you live? ___________________________
2. In what country were you born?
3. United States
4. Other:_______________
5. How many years have you lived

in the United States?

__ __ Years

1. What is your marital status?
2. Single
3. Partnered
4. Married
5. Widowed
6. Separated
7. Divorced
8. Other
9. What is your highest level of education? 1. 8^th^ grade or less
10. Some high school, but didn’t graduate
11. High school diploma or GED
12. Two year college degree
13. Four year college degree
14. Graduate degree

8. About how much is your yearly

household income? 1. $0-$5,000

1. $5,001-$10,000
2. $10,001-$15,000
3. $15,001-$20,000
4. $20,001-$25,000
5. $25,001 - $35,000
6. $35,001 - $45,000
7. $45,001 - $55,000
8. $55,001- $65,000
9. $65,001 - $75,000
10. $75,0001 or more

9. What type of dental insurance

do you have? 1. Medicaid or Title 19

1. Commercial or Private (ex. Delta Dental or

Blue Cross Blue Shield)

1. Dental Wellness Plan
2. None
3. Other_____________
4. Not sure/don’t know
5. There are many things that can cause problems to your teeth and your mouth. Which of the following items do you think can cause dental or mouth problems? Please circle **Yes (1), No (2), or Unsure (3)** to the following:

|  | **Yes** | **No** | **Unsure** |
| --- | --- | --- | --- |
| 1. People can inherit bad teeth/gums | 1 | 2 | 3 |
| 1. Having children takes the calcium out of teeth | 1 | 2 | 3 |
| 1. Eating a lot of sugar, candy, or snacks | 1 | 2 | 3 |
| 1. Bacteria | 1 | 2 | 3 |
| 1. General overall health affects the mouth | 1 | 2 | 3 |
| 1. Taking a lot of medications | 1 | 2 | 3 |
| 1. Drinking a lot of pop/soda/coke | 1 | 2 | 3 |
| 1. It is normal part of aging | 1 | 2 | 3 |
| 1. Not going to the dentist regularly | 1 | 2 | 3 |
| 1. Not brushing or flossing regularly | 1 | 2 | 3 |
| 1. Having a dry mouth | 1 | 2 | 3 |
| 1. Other: Please describe: | | | |

11. Overall, how would you rate the health of

your teeth and gums?

- - - 1. Excellent
      2. Very good
      3. Good
      4. Fair
      5. Poor
      6. Don’t know

1. When did you last brush your teeth?
2. This morning
3. Last night
4. I don’t brush my teeth
5. I can’t remember
6. Other:________________
7. When you brush your teeth,

what do you put on your toothbrush?

1. Toothpaste
2. Baking soda
3. Nothing
4. I don’t brush my teeth
5. Other:_________________________________________________
6. Which of the following resources do you use to get most of your information about oral and dental health? Circle **Yes (1) or No (2)**

|  | **Yes** | **No** |
| --- | --- | --- |
| 1. Radio/Television | 1 | 2 |
| 1. Internet | 1 | 2 |
| 1. Newspaper/Magazine | 1 | 2 |
| 1. Health newsletters/pamphlets | 1 | 2 |
| 1. Dentist/dental hygienist | 1 | 2 |
| 1. Physician/nurse | 1 | 2 |
| 1. Friends, relatives, neighbors | 1 | 2 |
| 1. WIC centers | 1 | 2 |
| 1. Headstart | 1 | 2 |
| 1. Health department | 1 | 2 |
| 1. Other: Please describe: | | |

1. If you had a toothache,

where would you go for treatment? 1. Emergency Room

1. Private doctor’s office
2. Private dental office
3. Community Health Center
4. University of Iowa College of Dentistry
5. Folk healer
6. I would use home remedies
7. Other________________
8. I don’t have a place to go
9. In which country do you normally

seek dental care? 1. Only in the U.S.

1. Only in my native country
2. Both equally
3. More in the U.S. than outside the U.S.
4. More outside the U.S. than in the U.S.
5. I don’t seek dental care
6. How long ago was your last visit

to a dentist, dental hygienist, or other

dental care provider? 1. Never

2. 6 months or less

1. More than 6 months, but not more than 1 year ago
2. More than 1 year ago, but not more than 2 years ago
3. More than 2 years ago, but not more than 5 years ago
4. More than 5 years ago
5. I don’t know
6. Which statement below best describes

when you go to the dentist?

1. I go to the dentist regularly (at least every 12 months)
2. I go only when I have pain or discomfort
3. I go only when I need something fixed
4. Other:____________

__________________

1. I have never been to the dentist-**Please stop survey**

If you answered, **“I have never been to the dentist”** in question 18, please **STOP** here.

**Thank you for your participation!**

If you have ever been to a dentist, please continue with the survey.

1. How important is it for you that your

dental provider speaks Spanish? 1. Very important

1. Somewhat important
2. Not important
3. How important is it for you that your

dental provider is of Hispanic/Latino

descent? 1. Very important

1. Somewhat important
2. Not important

Very important

1. If your dentist only speaks English, was a translator

provided to you during your last dental visit? 1. I didn’t need a translator

1. None present, but I wanted a translator
2. No, I had to bring my own
3. Yes, the entire appointment
4. Yes, part of the appointment
5. How often are you compliant with

attending your dental appointments? 1. Never (0%)

1. Occasionally (25%)
2. Sometimes (50%)
3. Often (75%)
4. Always (100%)
5. When you do not understand information

presented to you by your dentist, how

likely are you to ask questions? 1. Very likely

1. Somewhat likely
2. Not very likely

Please rate your agreement with the following statements. During my last dental appointment: **Strongly Agree (1), Somewhat Agree (2), Somewhat Disagree (3), or Strongly Disagree (4)**

|  | **Strongly Agree** | **Somewhat Agree** | **Somewhat Disagree** | **Strongly Disagree** |
| --- | --- | --- | --- | --- |
| 1. The dentist spent enough time with me. | 1 | 2 | 3 | 4 |
| 1. The dentist or staff treated me unfairly because of my race/ethnicity. | 1 | 2 | 3 | 4 |
| 1. The dentist or staff listened carefully to me. | 1 | 2 | 3 | 4 |
| 1. The dentist or staff explained things in a way I could understand. | 1 | 2 | 3 | 4 |
| 1. The dentist or staff showed respect for what I had to say. | 1 | 2 | 3 | 4 |
| 1. The dentist or staff asked me how I would like to learn about dental health. | 1 | 2 | 3 | 4 |

**The survey has ended. Thank you for your participation!**
